# Mapping Scientific Productivity Trends and Hotspots in Remdesivir Research Publications: A Bibliometric Study from 2016 to 2021

**DOI:** 10.3390/ijerph19148845

**Published:** 2022-07-21

**Authors:** Ropo E. Ogunsakin, Oluwakemi Ebenezer, Maryam A. Jordaan, Michael Shapi, Themba G. Ginindza

**Affiliations:** 1 Discipline of Public Health Medicine, School of Nursing & Public Health, College of Health Sciences, University of KwaZulu-Natal, Private Bag X54001, Durban 4000, South Africa; ginindza@ukzn.ac.za; 2 Department of Chemistry, Faculty of Natural Sciences, Mangosuthu University of Technology, Umlazi 4031, South Africa; ebenezer.oluwakemi@mut.ac.za (O.E.); jordaan.maryam@mut.ac.za (M.A.J.); mshapi@mut.ac.za (M.S.); 3 Cancer & Infectious Diseases Epidemiology Research Unit (CIDERU), College of Health Sciences, University of KwaZulu-Natal, Private Bag X54001, Durban 4000, South Africa

**Keywords:** bibliometrics, SARS-CoV-2, remdesivir, Scopus, science mapping

## Abstract

**Highlights:**

- 5321 articles are examined to study the literature on remdesivir research for the last six years.
- We use Scopus database to collect bibliographic material and provide a range of bibliometric indicators including the number of citations, publications, and authors.
- The analysis shows the highest influential articles and the most productive authors and their collaboration networks.
- The analysis shows which countries and institutions are leading research and the largest publishers.
- Co-word analysis was used to make a more comprehensive analysis of the development of the remdesivir research.

**Abstract:**

In response to global efforts to control and exterminate infectious diseases, this study aims to provide insight into the productivity of remdesivir research and highlight future directions. To achieve this, there is a need to summarize and curate evidence from the literature. As a result, this study carried out comprehensive scientific research to detect trends in published articles related to remdesivir using a bibliometric analysis. Keywords associated with remdesivir were used to access pertinent published articles using the Scopus database. A total of 5321 research documents were retrieved, primarily as novel research articles (*n* = 2440; 46%). The number of publications increased exponentially from 2020 up to the present. The papers published by the top 12 institutions focusing on remdesivir accounted for 25.69% of the overall number of articles. The USA ranked as the most productive country, with 906 documents (37.1%), equivalent to one-third of the global publications in this field. The most productive institution was Icahn School of Medicine, Mount Sinai, in the USA (103 publications). The *New England Journal of Medicine* was the most cited, with an *h*-index of 13. The publication of research on remdesivir has gained momentum in the past year. The importance of remdesivir suggests that it needs continued research to help global health organizations detect areas requiring instant action to implement suitable measures. Furthermore, this study offers evolving hotspots and valuable insights into the scientific advances in this field and provides scaling-up analysis and evidence diffusion on remdesivir.

## 1. Introduction

The pandemic of coronavirus disease 2019 (COVID-19), triggered by the recently evolving severe acute respiratory syndrome coronavirus 2 (SARS-CoV-2), has become an international health emergency [1,2,3]. This virus has spread globally with intense speed over a minimal period. According to researchers, this pandemic could continued for a more extended period; thus, several scientists across the globe are focused on exterminating the virus [4]. Although there is still no confirmed treatment for COVID-19, vaccines are under development, and several treatments have been proposed, while others are still ongoing [5,6]. Meanwhile, possible pharmacological treatments for COVID-19 may be found in broad-spectrum antiviral drugs, repurposed existing drugs or substances, and novel therapeutic agents [7]; among them, remdesivir has received particular consideration, according to the US Food and Drug Administration (FDA) [8]. Remdesivir is an inhibitor of the SARS-CoV2 RNA-dependent RNA polymerase (RdRp), which falls into the class of antiviral prodrugs established to treat infections caused by filoviruses and coronaviruses [9,10]. In the beginning, this drug was developed to treat hepatitis C [11,12] and, subsequently, it was considered for treating Ebola virus disease (EVD) [13] and Marburg virus infections [14,15,16]. However, in October 2020, the US Food and Drug Administration (FDA) approved remdesivir (GS-5734), and considered it a post-infection treatment for COVID-19. Considering the global desire for a potent and circumspect treatment for COVID-19 and the therapeutic potential of remdesivir, this study chose to analyze remdesivir in terms of its established inhibition of infection by the novel coronavirus in human cell lines [6,10].

According to a literature search on Scopus, more than nine hundred articles have been published on the use of remdesivir in COVID-19. Similarly, researchers have used different means to study the spread of COVID-19 within the short period of its invasion. However, data mining and statistical data analytic tools have not yet produced prominent procedures for the use of remdesivir in COVID-19. Thus, this study seeks to broaden the scope of existing studies on remdesivir in COVID-19 and addresses a gap in the literature by applying bibliometric analysis as a data-mining approach for the trends in the research on remdesivir to relate and analyze remdesivir-linked publications and citations, countries, and author impact. At the policy stage, bibliometric analysis has been recognized as one of the tools that can help decision makers to understand science and use it to discuss general issues. As a means of evaluating scientific productivity, bibliometrics can help create a data-driven picture of published scientific research and provide indication-based accounts and visualizations of research productivity.

Several studies have been conducted on remdesivir across the globe since the discovery of COVID-19. Prominent studies on remdesivir have applied the meta-analysis approach to reporting the pooled acceptance of remdesivir globally [17,18,19]. Most of these studies aimed to search for clinical evidence to support the use of remdesivir regarding its safety and side effects. Therefore, considering the number of published papers on this subject matter, investigators need to dedicate a substantial volume of time to reading and detecting appropriate work in interrelated disciplines considering the wide range of studies, the irregular quality of scientific research papers, and the considerable amount of data. Consequently, it is imperative to categorize substantial, active, and evocative evidence from massive databases to assist scientific investigations. Hence, the aim of this study is to help investigators to establish innovative research outlines and assist policymakers in appraising and recommending methods to explore study gaps. As the COVID-19 pandemic has not been entirely controlled and further information should be obtained from these references, bibliometric analysis is seriously needed. Hence, our study provides a timely and deeper understanding of the existing global literature on the use of remdesivir in COVID-19. With the increased global burden of infectious diseases, an increasing number of academic papers have been published. Evaluating the quality of such a significant number of research published papers and obtaining valuable information is imperative. Scientific research plays a vital role in understanding remdesivir. This bibliometric analysis highlights the features of the most influential published articles on remdesivir. It provides a comprehensive bibliometric analysis of the research on remdesivir, which will assist investigators in exploring potential research directions in this field. We expect to see further advances in the development of vaccines and therapeutics against infectious diseases targeting a broad spectrum of viruses to prevent future viral pandemics. Furthermore remdesivir research is continuously evolving; analyzing these changes will help researchers and scientific policymakers understand the status of remdesivir research.

## 2. Materials and Methods

In the current study, we deployed a bibliometric mapping analysis. Bibliometric mapping is a scientific discipline that uses a range of indicators to express the bibliographic characteristics of scientific publications quantitatively. Bibliometric analysis is a method used to describe publication patterns within a given field and is currently receiving increasing interest in many disciplines [20,21]. Bibliometrics analyses permits us to obtain deep insights from a moderately comprehensive study. Hence, it helps researchers to determine the contours of the research carried out on a definite topic.

### 2.1. Database and Search Approach

**Figure 1:**
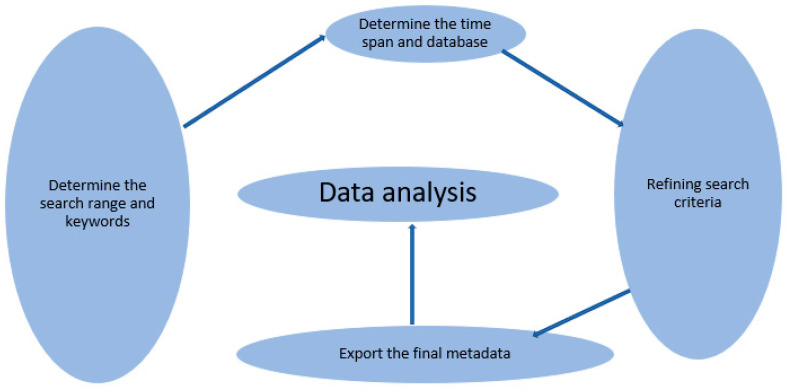
The methodological phase of bibliometric analysis.

The comprehensive literature published on remdesivir was extracted from the SCOPUS database. We performed a search on 3 December 2021. We searched the SCOPUS database for titles, abstracts, and keywords. The search terms included ‘remdesivir’ or ‘gs-5734’, ‘use of remdesivir’ or ‘gs-5734’. Scopus database was chosen because it is offers greater journal abstract and citation coverage compared with other databases, e.g., PubMed and Web of Science [22,23]. A total of 5321 publications were recovered after filtering based on the inclusion and exclusion criteria, Table 1. Subsequently, 2440 documents were eligible for the final bibliometric analysis. Comprehensive document lists were exported as BibTeX. Excel line charts, and the visualization maps were created with the help of biblioshiny interface. As suggested in our previous published paper, to analyze extracted data and results, it is essential to consider five vital phases that permit quantitative and qualitative analysis for bibliometric analysis [24]. Thus, the key theme of the study motivated the use of bibliometrics in the systematic review analysis of associated keywords. Figure 1 shows the bibliometric analysis process of this study, which mainly includes the following five phases: (1) determination of the search range and keywords; (2) determination of the time span and database; (3) refining of search criteria; (4) export of the final metadata into the visualization software; (5) data analysis. As a result, the fundamental objective of this research was to link studies from various years, countries, and journals to the same research parameters.

### 2.2. Bibliometric Methodology and Statistical Data Analysis

Bibliometrics is a form of library and information-sciences study that examines bibliographic material using quantitative methods [24]. Bibliometrics is extensively used for abridging the maximum illustrative results of a set of bibliographic documents. In addition, it highlights the performance of authors and institutions and their impact on scientific productivity. Furthermore, the approaches to bibliometric analysis show two classifications: (i) performance analysis and (ii) science mapping. First and foremost, the performance analysis caters for the impact of research constituents, which is descriptive, but the hallmark of bibliometric studies. Specifically, it uses various markers to evaluate the effect of citations of authors/institutions/countries on scientific production. Meanwhile, the number of publications and citations and the *h*-index are the widest markers used for analysis.

In a different vein, science mapping or bibliometric mapping focuses on the relationships between research constituents. These two approaches can be incorporated to analyze bibliographical evidence with validation upon developing various bibliometric analysis software/tools. The current study deployed bibliometrix R-package software, open-source software intended to for use in quantitative scientometrics and informetrics. It has the key algorithms for performing statistical analysis and science-mapping analysis. The recent versions of the bibliometrix R-package contain a web interface app (Biblioshiny) to help users without coding skills conduct bibliometric analysis. Biblioshiny also permits users to achieve appropriate bibliometric and visual examinations on an interactive web interface, significantly reducing their data-input skill requirements. Biblioshiny interface allows importing of data from Scopus or Web of Science databases in BibTex format. It also helps to filter data in biblioshiny. Thus, our study exploited these opportunities inherent in biblioshiny for bibliometrix to import data from WoS in BibTex format. Hence, the bibliometrix and biblioshiny packages were deployed for this paper’s analyses. This package uses the meta-data in Scopus to calculate and rank country production, journal sources, and country collaborations. Drawing on inferences from the previous bibliometric, the national production was defined using the first author’s country.

## 3. Result

### 3.1. Spatial Distribution of Publication Information

On 3 December 2021, a total of 5321 research documents on remdesivir, largely as novel research articles (*n* = 2440; 46%), were retrieved from the SCOPUS database with a timespan of 2016–2021 (Table 1). This implies that the original research articles were the primary type. The preliminary information detailed that the documents were from 971 sources. The average year from publication was 0.411, including 89,700 references, whereas the average citations per document and average citation per year per document were 24.04 and 13.33, respectively. The document contents included over 13,586 keywords (ID) and 4046 author keywords (DE). The results showed there were 22,515 authors; 119 authors published single-authored documents, and 22,396 authors published multi-authored documents. The annual publication flow from 2016–2021 detailed that the number of documents (five articles) per year was maintained from 2016–2018. In 2019, the number of articles increased to nine, indicating researchers’ slight interest in remdesivir.

Interestingly, the documents considerably increased to 926 in the year 2020, indicating significant findings in this year. Furthermore, there was still a steady increase in records in the year 2021 (1492), which was the highest number of records per year to date due to the significant discoveries on remdesivir. The average number of citations per year was analyzed by observing the number of citations of remdesivir documents. This result showed the influence that publication has on the field annually. The result indicated that the documents for 2016, 2017, and 2018, which appear to constitute the beginning of the field, received average total citations (TCs) of 41.8, 73.55, and 59.1 per year, respectively. The average citations continued to increase for 2019 (79), dropping sharply to 48.57 in 2020.

### 3.2. Most Local Citations and Sources’ Local Impact

The results of the top 20 sources with the most local citations, based on the cited references and the top 20 sources with the greatest local impact that focused on remdesivir articles, are presented in Table 2. The local-impact sources were measured using the *h*-index. The *h*-index is also called the *h*-factor, which stands for high citations. Hirsch proposed the *h*-index in 2005. It is used for the academic evaluation of researchers [25]. The parameter is consistent and accurate in measuring an individual’s scientific achievements. However, although the index was initially developed for the analysis of individuals, it has also been applied at other levels, including research groups, departments, and institutions [26]. In particular, the *h*-index may differ based on the database used to calculate it, as the database may index a different number of articles from each source.

The journal ranked first for the highest number of local cited sources was the *New England Journal of Medicine*, with 3833 articles, followed by *The Lancet*, with 2679 articles. The third and fourth journals were the *American Medical Association* and *Nature*, with 1978 and 1658 articles, respectively. Other heavily cited sources, according to the assessment, included the *Journal of Virology* (1107), *Science* (997), *Clinical infectious disease* (839), *Cell* (821), and *Journal of Medical Virology* (764). Notably, the journal was ranked number 17 for both number of local cited sources and local source impact (*h*-index = 7, *m* index = 20, TC = 455, respectively). In comparison, for local source impact, *Clinical Infectious Disease* and *Journal of Biomolecular Structure and Dynamics* took the third and fourth positions, respectively, with TCs of 704 and 609 and *h*-indexes of 11 and 10, respectively. Furthermore, *Nature* moved from the fourth to the seventh position in terms of local impact; meanwhile, the *American Journal of Transplantation* was ranked number two, and *Clinical Infectious Diseases* was number four. Another local source of impact was ‘PLoS ONE’, previously *PLoS ONE*, an open-access, peer-reviewed scientific journal published by the Public Library of Science (PLoS) since 2006. The journal publishes multi-disciplinary and interdisciplinary topics. *PLoS ONE* aims to publish quality research in more than 200 fields, including science, engineering, medicine, and associated social and human sciences. The journal was ranked number six based on the dataset used for the analysis, with an *h*-index of 10. Furthermore, the only journal from the Frontiers Media SA publisher that was ranked in the top 20 for greatest local impact was *Frontiers in Pharmacology*, with an *h*-index of 8 and a TC of 132.

### 3.3. Most Local and Global Cited Documents

This section details the most locally cited and globally cited documents. The top 20 most frequently locally cited (LC) and globally cited (GC) articles selected from 2016 to 2021 are listed in Table 3 and Table 4. Local citation refers to the number of citations an article obtained from articles in the analysis data, emphasizing only the subject under review. Global citation refers to the number of citations an article received from the whole database. It quantifies the influence of an article, which, in most cases, can receive its most significant number of citations from other subjects. Remarkably, the higher the LC, the more influential the article is on remdesivir research and further research under investigation. Notably, the number of citations does not necessarily reflect the quality of an article, but it is a quantifier of its impact and visibility in the research area. The paper published by Holshue ML et al. [27] titled ‘First case of 2019 novel coronavirus in the United States’ was the most frequently globally cited article, with a TC of 2997 and a value of 1498.5 total citations per year. The next most globally cited article was the paper published by Beigel JH et al. [28], titled ‘Remdesivir for the treatment of COVID-19—Final Report’, published in 2020 by the *New England Journal of Medicine*, which also appeared to be the most frequently globally cited article; the article was cited 2458 times. The authors believed that remdesivir could shorten the recovery period by reducing the level of respiratory tract infection compared to the reference drug in adults hospitalized with COVID-19. The paper titled, ‘Remdesivir in adults with severe COVID-19: a randomized, double-blind, placebo-controlled, multicenter trial’, written in 2020 by Wang et al. [29], was cited 1575 times after it was published in *Lancet* and was ranked third on the list. The authors reported the results of a placebo-controlled randomized trial of remdesivir in patients with severe COVID-19. The trial was conducted at ten hospitals in Wuhan, Hubei, China. Their results showed that the intravenous remdesivir regimen was appropriately tolerated but did not have significant clinical or antiviral effects in critically ill patients with COVID-19.

Meanwhile, the paper titled ‘Compassionate use of remdesivir for patients with severe COVID-19’ also had the highest contribution of articles among the 20 most globally cited articles, demonstrating its predominance. The local citation results detailed that the studies published by Wang Y, Grein J, Sheahan TP, Warren TK, Agostini TK, Holshue ML, Goldman JD, Mulangu S, De Wit E, and Gao Y were among the 20 most frequently globally and locally cited articles, respectively. Notably, Berlin Da et al.’s study was ranked number one in most local citations. The article had a local citation value of 940 and a global citation value of 398. Meanwhile, Wang Y et al.’s article was ranked second among the most frequently locally cited articles, and Grein J’s was ranked third. Notably, the published work of Berlin DA received more local citations than global citations.

### 3.4. Word Cloud of the Most Frequent Keywords Plus

**Figure 2:**
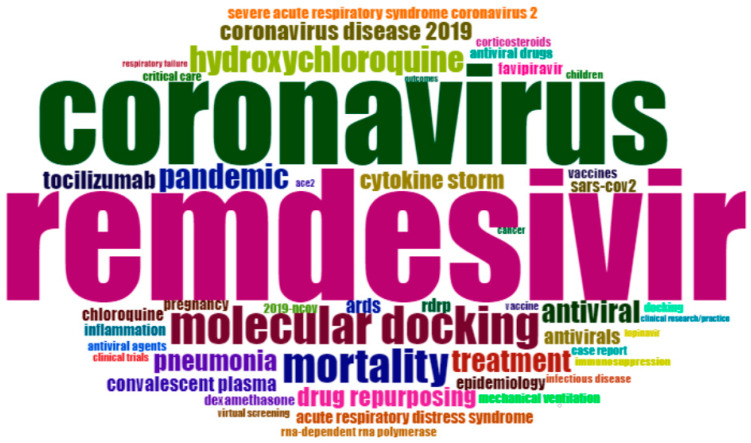
Word cloud of author keywords. The word cloud describes the frequency of author keywords in remdesivir research (**Source:** Author calculation).

Figure 2 shows the result of the word-cloud analysis made from the authors’ keyword. The figure depicts the most populated areas, such as COVID-19, SARS-CoV-2, remdesivir, coronavirus, molecular docking, mortality, hydroxychloroquine, pandemic, treatment, antiviral, etc. The word cloud is dominated by ‘COVID-19’ (1324), ‘SARS-CoV-2’ (750), ‘remdesivir’ (326), ‘coronavirus’ (250), ‘molecular docking’ (94), ‘mortality’ (86), ‘hydroxychloroquine’ (65), ’pandemic’ (62), ‘treatment’ (57), and ‘antiviral’ (56), which suggests that these terms have the highest frequency in the literature on remdesivir. Another frequent keyword is ‘pneumonia,’ which is strongly associated with coronavirus [54,55]. This is why much research is conducted on the therapeutic efficacy of remdesivir. Many researchers link remdesivir with drug repurposing [56] and rdrp [57]. All the keywords were interrelated and addressed many current issues.

### 3.5. Co-Occurrence-Network Analysis Using Keywords and Keywords Plus

**Figure 3:**
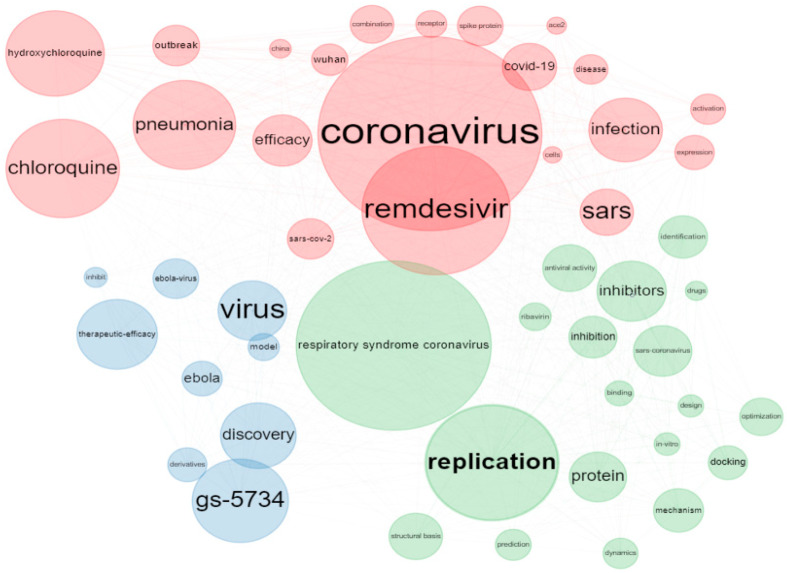
Keyword-co-occurrence-network visualization for remdesivir research.

Figure 3 shows the keyword-co-occurrences network for the remdesivir research. The co-occurrences of author keywords are divided into two groups. The size of the nodes in the keyword-co-occurrences network depicts the degree of author-keyword co-occurrence, which represents the number of times two keywords occur together [24]. Thus, the bigger the node, the higher the relevance of the keywords. The lines between the nodes represent the keyword frequency, while the different node colors represent other clusters. The links between nodes define the relationships.

Furthermore, the authors’ keywords and keyword-plus distribution were evaluated to find the most common search topics and their preferences. The analysis of the authors’ keywords provides evidence of research trends from the perspective of researchers and has proved crucial for the development of science [58]. Meanwhile, keywords plus provide additional search terms taken from the article titles quoted by authors in their bibliographies and footnotes [59]. In addition, cluster analysis can be used to explore the themes of articles derived from keywords and keywords-plus of authors; thus, co-occurrence-network analysis was utilized to gain insights about trends n remdesivir research.

### 3.6. Dissemination of Author Keywords

**Figure 4:**
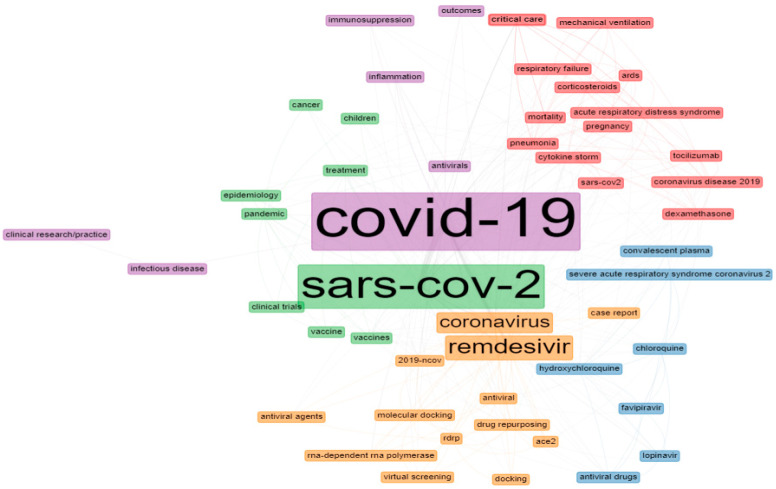
The top 50 author keywords in documents related to remdesivir. **Note:** pink—cluster 1, blue—cluster 2; green—cluster 3; purple—cluster 4; orange—cluster 5.

The analysis of the author keywords from this study period discovered 4046 author keywords. The author keywords in articles that referred to remdesivir were evaluated, and the top 50 author keywords were used and clustered from 2016 to 2021 (Figure 4). The node and word size depict the nodes’ weight, while the spacing between the nodes indicates the intensity of the relationship between them. The lines between the keywords highlight that they appeared simultaneously; the thicker the line, the greater the co-occurrence. Nodes with the same color are grouped. The top three most frequently used keywords were ‘SARS-CoV-2’, ‘COVID-19’, and ‘remdesivir’, which was in strong agreement with the research trend.

The 50 author keywords were divided into four groups and represent the major research areas on remdesivir; the first cluster included ‘mortality’, ‘drug pneumonia’, ‘coronavirus disease 2019’, ‘cytokine storm’, ‘tocilizumab’, ‘ards’ ‘SARS-CoV-2’, ‘acute respiratory distress syndrome’, ‘pregnancy’, ‘dexamethasone’, ‘mechanical ventilation’, ‘corticosteroids’, ‘critical care’, and ‘respiratory failure’. For a critically hospitalized COVID-19 patient, severe pneumonia can lead to acute respiratory distress syndrome (ARDS) or respiratory failure, associated with cytokine-storm syndrome. Tocilizumab and remdesivir have been reported to treat severe COVID-19; however, additional data are necessary to guide risk–benefit considerations [60,61,62,63]. Jo et al. stated that using remdesivir for unventilated patients and dexamethasone for ventilated patients may be inexpensive compared to standard care by reducing the number of days patients spend in intensive care [64].

The second cluster included ‘hydroxychloroquine’, ‘chloroquine’, ‘favipiravir’, ‘antiviral drugs’, ‘lopinavir’, ‘severe acute respiratory syndrome coronavirus 2’, and ‘convalescent plasma’. These results reflected the reported research on anti-inflammatory effects and in vitro studies proposing the antiviral activity of hydroxychloroquine. Self et al. [65] conducted a randomized clinical trial to evaluate hydroxychloroquine’s efficacy in treating adults hospitalized with COVID-19 (NCT04332991). The authors included remdesivir in the post hoc analyses among subgroups of patients treated clinically with other open-label drugs. Their results do not support using hydroxychloroquine to treat COVID-19 inpatients due to the minor improvement observed in the patients’ clinical condition.

It should be noted that large multinational pharmaceutical companies play a crucial role in the fundamental research on the use of remdesivir for treating or preventing COVID-19. Remdesivir and chloroquine phosphate monotherapy inexpensively reduce SARS-CoV-2 infection. Meanwhile, remdesivir is a nucleoside analog prodrug developed by Gilead Sciences, a biopharmaceutical company in the USA [27,35,48,63,66,67,68]. Furthermore, during the pandemic, many researchers evaluated the use of combination therapy to fight the spread of COVID-19 [69]. Combination therapy consisting of remdesivir with chloroquine, ivermectin, or doxycycline has been reported. The combination of remdesivir and ivermectin has shown powerful synergy in achieving significant reductions in the cytokine levels of IL-6, TNF-α, and leukemia-repressing factor. Furthermore, the combination of remdesivir with doxycycline reduces virus levels. The authors suggested further studies based on their findings to explore the mechanisms of action of combination therapy, in vivo experiments, and clinical trials in the treatment of SARS-CoV-2 infection [70]. Baricitinib, in combination with remdesivir, has been reported to be superior to the monotherapeutic use of remdesivir to reduce recovery times and accelerate clinical-status improvement for COVID-19 patients [71], particularly among those receiving high-flow oxygen or noninvasive ventilation. However, the combination had a less severe effect. So far, the antiviral activity of remdesivir has been shown to be superior compared to other tested drugs on COVID-19. The third cluster included ‘SARS-CoV-2’, ‘pandemic’, ‘treatment’, ‘antiviral’, ‘epidemiology’, ‘inflammation’, ‘vaccines’, ‘children’, ‘clinical trial’, ‘vaccine’, and ‘cancer’. The fourth cluster included ‘COVID-19’, ‘antivirals’, ‘inflammation’, ‘immunosuppression’, ‘infectious disease’, ‘clinical research/practice’, and ‘outcomes’. The fifth cluster included ‘remdesivir’, ‘coronavirus’, ‘molecular docking’, ‘antiviral’, ‘drug repurposing’, ‘RdRp’, ‘2019-nCoV’, ‘antiviral agents’, ‘RNA-dependent RNA polymerase’, ‘case report’, ‘docking’, ‘virtual screening’, and ‘ACE2’.

To suggest possible COVID-19 inhibitors to reduce the spread of the virus, many researchers explored computer-aided drug design (CADD), including pharmacophore modeling and molecular dynamics, as well as quantitative structure–activity relationship (QSAR), molecular docking, quantum mechanics ADMET, and virtual screening for drug repurposing that can target COVID-19 [72,73,74]. Elfiky AA [75] analyzed the drugs currently on the market or in clinical trials that inhibit COVID-19 using a molecular docking approach. Among the 24 drugs repurposed using molecular docking studies, ribavirin, remdesivir, sofosbuvir, galidesivir, and tenofovir showed promising results against the newly emerged strain of coronavirus. Meanwhile, setrobuvir and YAK compounds effectively bind to the amino acid in the active site of SARS-CoV-2 RdRp. In the research work of Naik et al., ten antiviral drugs were docked in the binding site of the main SARS-CoV protease to understand their effectiveness against 2019-nCoV. Remdesivir showed an excellent docking score, with solid binding affinity and steady confirmations with the crucial residues, Cys145 and His164, of the main SARS-CoV protease, with a binding affinity of 8.2 kcal/mol, which inhibited the replication and proliferation of 2019-nCoV [76]. Wu et al. screened their database, the Food and Drug Administration (FDA)-approved drugs in the ZINC database, and a database of commonly used antiviral drugs. Among the docked compounds, remdesivir showed excellent binding in the active pocket of SARS-CoV-2 RdRp, and human TMPRSS2, a protein facilitating infection with the virus. Human ACE2 was among the protein targets, although several compounds were observed to bind with ACE2 protein through virtual screening. However, none of the compounds bound at the contact surface of the ACE2-spike complex, signifying that these compounds are only ACE2 enzyme inhibitors rather than ACE2 viral infection inhibitors [34]. Notably, in the rapidly advancing pandemic, repurposing drugs in clinical trials and assessing commercially available and accessible inhibitors against the druggable targets of SARS-CoV-2 has been a helpful approach that has enhanced the drug-discovery process.

### 3.7. Dissemination of Keywords Plus

The top 50 common keywords plus and the co-word networks were analyzed and visualized using the biblioshiny interface. The result of the keywords-plus analysis showed that the top four most frequently used were ‘coronavirus’, ‘remdesivir’, ‘respiratory syndrome coronavirus’, and ‘replication’. Similarities between the author keywords and the keywords plus were observed Similarly to the results of author keywords, ‘coronavirus’, ‘remdesivir’, ‘sars’, ‘chloroquine’, ‘COVID-19’, ‘hydroxychloroquine’, ‘disease’, ‘SARS-CoV-2’, ‘cells’, ‘activation’, ‘expression’, ‘receptor’, ‘combination’, ‘spike’, ‘protein’, ‘gs-5734’, ‘ebola’, ‘therapeutic efficacy’, and ‘discovery’ also featured in the top 50 commonly used keywords (Figure 4). Moreover, the prevalence of ‘replication’, ‘ribavirin’, ‘antiviral activity, and ‘ebola-virus’ demonstrate that research on viral diseases has been a major topic in recent years—notably, research interests associated with Wuhan and China garnered more attention. Furthermore, there was a growing interest in research on ‘outbreak’, as well as in topics related to ‘efficacy’, ‘infection’, and ‘pneumonia’, based on the ranking of these keywords.

### 3.8. Academic Collaboration

**Figure 5:**
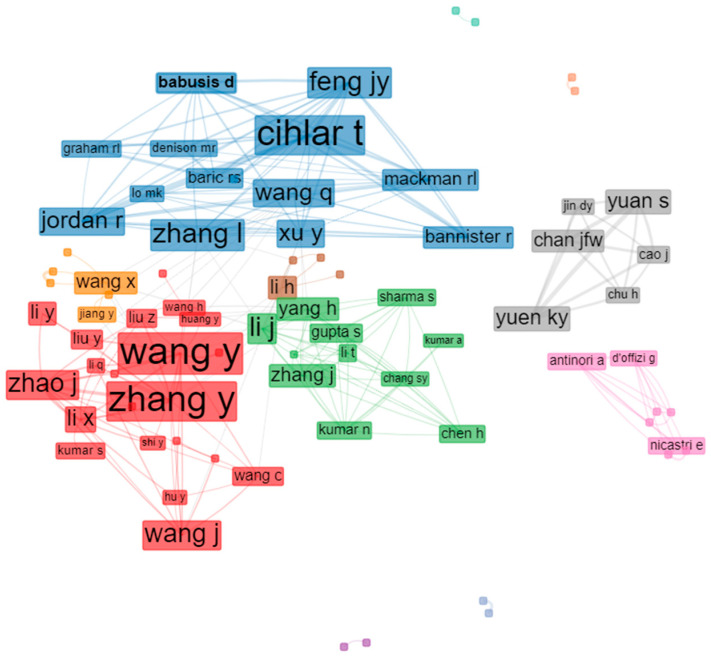
Visualization of academic collaboration between the authors.

Academic collaboration between authors usually greatly facilitates knowledge and exchange, thereby broadening the field in question. As expected in the remdesivir research field, notable cooperative relationships occurred at multiple levels (Figure 5). To identify the authors who contributed the most, we ranked them by their total number of citations. Based on the data extracted, we found that Wang, Y., and Zhang, Y. ranked first in terms of co-citations. The two authors received the most recognition in this field and made outstanding contributions. The cooperation-network visualization was performed using the biblioshiny interface. In the collaboration network among authors, institutions, or countries, the Louvain method was used as a clustering algorithm; the number of nodes used was 50, and the minimum edges used was 2 to avoid isolated and one-time collaborations; meanwhile, the isolated nodes were removed. The author-collaboration map reflects the scientific-research cooperation between the authors. The rectangle/node signifies the authors; the size of the circle/node signifies the number of articles. The lines denote the authors’ collaboration strengths, and each color represents a cluster. Notably, the thickness of the connections between the nodes displays the collaboration frequency.

### 3.9. Most Frequently Cited Countries and Country’ Collaborations

**Figure 6:**
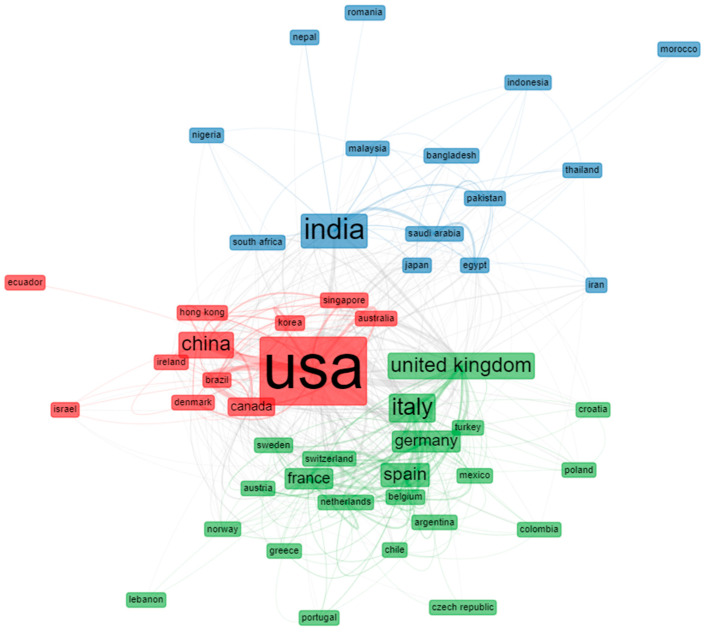
Visualization of collaborative network by countries.

The most cited countries, with their total citations and average article citations, are shown in Table 5. Interestingly, the United States, China, France, and India were the four countries with the highest number of citations, with average article citations of 39.82, 50.92, 50.78, and 14.91, respectively. In addition, assessing the state of collaboration by examining inter-institutional partnerships is very informative. The country-collaboration network of remdesivir-related articles is shown in Figure 6. The network reveals the level of idea exchange between the countries and the dominant countries in this field. The four different colors on the map imply the broadening of research trends. Notably, the significant nodes epitomize the leading countries, while the connection between the nodes signifies the institutional relationships. Moreover, the space between the nodes and the thickness of the connections mean the intensity of collaboration between the countries.

Figure 6 shows the collaborative network flanked by countries researching remdesivir. The thicker the link between the countries, the stronger the collaborative relationship, and vice versa. The closest relationships in this collaboration network were between the USA, the United Kingdom, Italy, India, and China. The United States led the largest group, not limited to its geographical area. It collaborated with European and Asian countries and with neighboring countries, such as Canada. India led the second group, working closely with Saudi Arabia and Pakistan. Furthermore, it could also be seen as one of the focal points of the entire network in international collaboration. The third group, headed by Italy, had the same characteristics as the USA. Italy had a collaborative network with the United Kingdom and Spain and strong collaborations with European countries, such as Belgium, Spain, Germany, and the Netherlands.

### 3.10. Most Relevant Affiliations and Institutional Collaborations

The contributions from and collaborations between the various institutes were evaluated by the institutes’ affiliations, with a minimum of one author for the documents. The 20 highest-ranking institutions with over 20 papers were ranked by their documents. Table 6 shows that Icahn School of Medicine, Mount Sinai, in the USA, was the most influential institution in the study of remdesivir. The institution published 103 articles related to remdesivir and was ranked first, followed by Harvard University (81 articles), University of California (67 articles), University of Michigan (53 articles), and University of Washington (49 articles). At the same time, Gilead Science, a research-based biopharmaceutical company, is a prominent company in terms of article quality.

**Figure 7:**
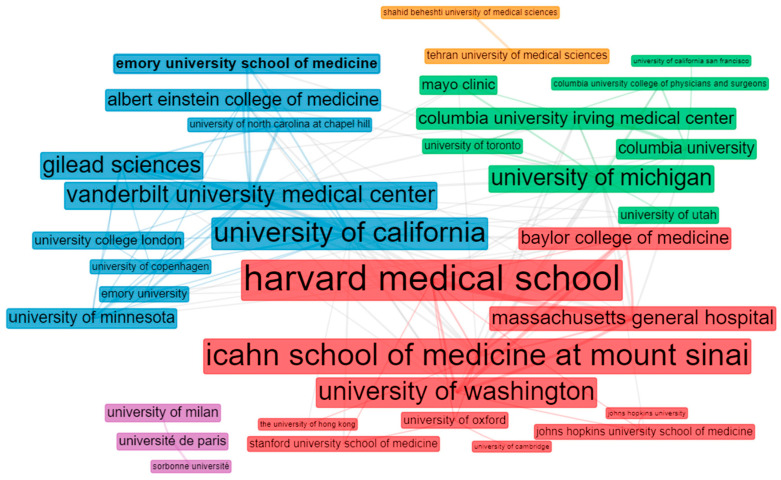
Visualization of institution collaborations.

Meanwhile, five clusters are detailed in the institution-collaboration pattern from the 50 most productive institutions. The two largest groups comprise institutions in the USA. Icahn School of Medicine at Mount Sinai, Vanderbilt University, University of Washington, University of Michigan, Harvard Medical School, and the University of California were the central sub-groups, respectively. The Gilead Science biopharmaceutical company acted as a bridge for increasing the collaboration among these institutions. Thus, the USA institutions and the Gilead Science organization formed a core between the corresponding poles (Figure 7).

The analysis showed two main trends of documents related to remdesivir. The first was clinical studies to evaluate remdesivir at the individual level regarding patients suffering from COVID-19, the repurposing of remdesivir for further applications, mono/combination therapy, and clinical-trial outcomes. This promoted a better understanding of remdesivir’s clinical effectiveness, which helped clinicians develop modified therapeutic strategies. The second was computer-aided drug design, including pharmacokinetics or immunoinformatics, which also paved the way for remdesivir to control the COVID-19 pandemic. Although we achieved exciting results with the help of bibliometric analysis and the visualization of documents related to remdesivir, this study has some limitations. The analyzed documents were downloaded from a single database (Scopus), and the articles were written in English, leading to the underestimation of researchers who used other languages.

## 4. Discussion

### Synopsis of Evidence

Remdesivir is a broad-spectrum antiviral medication, which was initially proposed to treat the Ebola outbreak [8]. The global pandemic drove the biomedical community to uncover and develop antiviral interventions, following evaluation by various virology laboratories. Consequently, the COVID-19 pandemic led to many scientific publications in remdesivir research. This led to the establishment of the ability of remdesivir to inhibit COVID-19. Therefore, the current state of remdesivir research needs a comprehensive global analysis to help guide plans for future study, especially through collaborations between various academic researchers in various fields. As a result, this study’s goals were to analyze publication trends, the most productive journals, the most productive authors, and the most relevant keywords and countries in the field of remdesivir research. Consequently, the current study offers a global bibliometric analysis of remdesivir research. Thus, the findings emanated from this study may contribute to further theoretical study. Based on the previous studies, the number of articles published over the years can reflect productivity and development [77,78].

The findings from the data gathering indicate that a total of 5321 documents linked to remdesivir research were published in the Scopus database between 2016 and 3 December 2021. The outcomes of our study revealed that from 2016 to 3 December 2021, the number of publications on remdesivir grew steadily. The increasing trend in the number of related publications suggested that researchers were increasingly focused on remdesivir. Furthermore, the distribution of the documents revealed that published articles (*n* = 2440; 45.86%) were the largest category, followed by reviews (*n* = 1566; 29.43%).

In addition, most of the documents in the remdesivir research between 2016 and 2018 had an average TC of 41.8, 73.55, and 59.1 per year, respectively, while the average citations increased to 79 in 2019 and dropped sharply to 48.57 in 2020. Furthermore, the most locally and globally cited journal in remdesivir research was the *New England Journal of Medicine*, followed by *The Lancet*. In addition, our study showed that the number of publications on remdesivir fluctuated during the year under investigation. This observation was related to findings in previous bibliometric analyses in the literature [24,79]. However, when the number of publications on remdesivir was presented for the year 2020, it was apparent that there was an overall increase in the number of publications in that year. It is expected that the rate of publication on remdesivir will increase with time.

Moreover, from a national point of view, the analysis demonstrates how remdesivir research developed in countries located on different continents over the past six years. The USA and the United Kingdom were enormously active compared to the African countries. The results also suggest that the USA significantly dominated in all scientific publications. In addition to the USA, which substantially outperformed other countries, China and Italy dominate in remdesivir research. Moreover, in terms of collaboration, the USA collaborated more than any other country, based on our findings emanated. It collaborated with European and Asian countries and with neighboring countries such as Canada. This implies that these countries invested substantial funds, human resources, and material resources in scientific research. Therefore, it is no surprise that they become world leaders in the use of remdesivir, particularly as other bibliometric studies have established similar findings [80]. At the same time, the results also reveal that the United Kingdom, India, China, and the United States collaborated the most closely because of the close academic exchanges between researchers in the two countries, and overseas researchers continued to cooperate within the framework of the international network. However, considering the number of citations and *h*-index, the United Kingdom was the most influential. The implication is that publishing documents in diverse countries may enhance a country’s significance and impact with regard to remdesivir research. Furthermore, the USA ranked highest in terms of citations (*n* = 26,602), followed by China (*n* = 7230) and France (*n* = 3047), and the frequency of publication varied among the most prominent countries.

Further, the findings revealed that most of the countries were related through the lines indicated signifying the collaboration networks between the countries on the network map. The global focus on remdesivir research was demonstrated by the high *h*-index value, which indicated that there were many readers and citations on the topic. Another indicator of the worldwide focus on remdesivir was the most-cited articles on remdesivir research. Regarding the most relevant institutions and organizations, the results also suggested that Icahn School of Medicine and Mount Sinai in the USA played crucial roles in remdesivir research. Meanwhile, the leading partner institution was Gilead Science, a research-based biopharmaceutical company that conducted a long-term study on the remdesivir. The most influential authors of remdesivir research were Wang, Y. and Zhang, Y. Furthermore, the results of the keyword co-occurrence analysis revealed diverse areas of research focus for individual scientific researchers. Overall, the two most featured words were ‘coronavirus’ and ‘remdesivir’. As explained previously, in the keyword co-occurrence networks, the circle size represented the number of articles in which an author keyword was found. However, the strength of the link denoted the number of times the two keywords were found together. Groups of nodes sharing a high number of co-occurrences were clustered together and colored accordingly for each bibliometric network [28].

Accordingly, this study comprehensively investigated scientific progress on remdesivir using bibliometric analysis techniques. With regard to the collaboration status of remdesivir research globally, more interdisciplinary, multi-institutional, and global research collaborations are needed to achieve critical breakthroughs in this research area. This systematic bibliometric analysis, to our understanding, may be an initial phase in the tracking of the progress and prevalence of remdesivir research. However, restrictions relating to the study need to be stated, which have also been revealed in previous bibliometric published studies. The first limitation is that the data deployed for the analysis were retrieved from only one database: Scopus. Unfortunately, this may not cover the full range of the literature because some journals are not indexed in Scopus. Finally, due to the urgency of the COVID-19 epidemic, a large volume of research has been published as in pre-print form in several archives. Using Scopus alone limited accessing the pre-prints in these archives. Therefore, including other databases, especially the expanding body of pre-prints available in the Google Scholar database, could have provided additional insights not available in this study. Another limitation observed in this study was the time frame of six years (2016–3 December 2021). This limitation could not be addressed in this study; hence, a systematic study with a longer time frame would yield further time-dimensional insights. This would also be beneficial in terms of achieving a higher number of publications. Finally, considering only the titles, abstracts, and keywords in English as an inclusion criterion can lead to publication bias. Thus, future studies should address this issue.

In light of the results, future bibliometric studies should address these limitations and further examine the evolution of remdesivir research. Despite these limitations, the data offered by this database include the overwhelming majority of the publications in remdesivir research. In addition, we tried our best to corroborate the data by manual review and to perform a comparative global evaluation of remdesivir research productivity trends to produce a comprehensive overview of remdesivir research. Nevertheless, the findings of this study highlight the importance of a comprehensive and in-depth approach that uses systematic reviews and bibliometric analyses in remdesivir research. Additionally, we analyzed the scientific impact of the twenty highest-ranking countries, journals, and institutions, and not all the data. In this way, we ensured the validity of the data through manual review. We attempted to present a close overall assessment on remdesivir research productivity that offers a helpful supplement to the literature on remdesivir.

## 5. Policy Implications

Since the identification of the infectious coronavirus disease, many scientific publications have been produced locally and globally. The current remdesivir research analysis offers a wide-ranging mapping of the research trends and productivity related to remdesivir through documents indexed in the Scopus database. At the same time, it provides the reader with wide-ranging evidence on the research productivity and insight into remdesivir’s research features. The findings from this systematic bibliometric analysis have significant policy implications for evaluating and monitoring the scientific research output on remdesivir. First, mapping the research trends related to remdesivir is essential to assist countries in designing appropriate interventions to inform vulnerable people and reduce pressure on health systems. This evidence can also support a wide-ranging evaluation of social and economic implications. This will offer vital information for developing surveillance policies to address health issues in regions with low research output and will shed light on possible future collaborations and potential joint research activities. Similarly, the results of the bibliometric study offer a baseline level of activity and performance and provides a set of probable indicators whose use may be pragmatic in future analyses of the research on remdesivir. Moreover, the robust bibliometric methods in this study may offer a more vigorous picture of the field beyond traditional citation analyses.

## 6. Conclusions

In this paper, we analyzed the bibliometric features of highly cited articles in remdesivir research. The advancement in remdesivir research is recognized given the total number of publications in the Scopus database. A total of 2440 frequently cited articles were found, covering the period between 2016 and 3 December 2021. Our study showed that the rate of publication on remdesivir research gained momentum from 2020. Publications on this topic appeared in high-impact journals, indicating the global c dimension of the remdesivir issue. The articles had high numbers of citations and *h*-indexes, indicating readability and quality. The *New England Journal of Medicine* was the most productive journal, with an *h*-index of 13. The Icahn School of Medicine, Mount Sinai, in the USA, also contributed 103 articles, accounting for 4.22% of the total. The USA, the United Kingdom, Italy, India, and China were significant contributing countries. Most of the twenty most frequently cited articles were published in the *New England Journal of Medicine*. Moreover, the institutions in the USA had a higher share of publications on remdesivir. International collaboration is of significant value and can boost the volume and scientific impact of publications on remdesivir, predominantly in countries with inadequate resources. Thus, this study helps the scientific community to identify the leading journals, authors, research institutions, and countries of remdesivir research publications. Similarly, this paper could be help scientists and researchers to understand the achievements and developments in remdesivir research. Furthermore, administrators and policymakers in countries with low research output should consider increasing the support for remdesivir research to generate knowledge that can be used to curb the global pandemic. 

Through a bibliometric visualization analysis, visually presented the key areas of progression in remdesivir research. Furthermore, the findings in this study provide a helpful reference for medical virologists, epidemiologists, policy decision makers, academics, and infectious-disease-vaccine researchers. On the other hand, due to the sparsity of hierarchical cluster analyses of published remdesivir articles, a hierarchical cluster analysis was not adopted in this study. In the future, studies based on hierarchical cluster analysis will be conducted and included in our further analyses.

## Figures and Tables

**Table 1:** Types of retrieved documents on remdesivir research publications (2016–2021).

Type of Document	Frequency (*n*)	%
Article	2440	45.86
Review	1566	29.43
Letter	438	8.23
Editorial	252	4.74
Note	251	4.72
Short survey	46	0.86
Book chapter	31	0.58
Erratum	15	0.28
Conference paper	14	0.26
Data paper	1	0.02
Retracted	2	0.04
Trade journal	3	0.06
Non-English article	80	1.50
Irrelevant documents	182	3.42

**Table 2:** Top 20 most local cited sources and source local impact.

Journal	Greatest Local Source Impact				Most Local Citations	Articles
	*h*_Index	*g*_Index	*m*_Index	TC		
*New England Journal of Medicine*	13	14	3.30	10,997	*New England Journal of Medicine*	3833
*American Journal of Transplantation*	12	22	6	856	*Lancet*	2679
*Journal of Biomolecular Structure and Dynamics*	11	26	5.5	704	*The Journal of the American Medical Association*	1978
*Clinical Infectious Diseases*	10	14	5	609	*Nature*	1658
*European Journal of Pharmacology*	10	17	5	318	*Journal of Virology*	1107
*PLoS ONE*	10	14	5	217	*Science*	997
*Nature*	9	9	1.5	1681	*Clinical Infectious Disease*	839
*Antimicrobial Agents and Chemotherapy*	8	15	4	396	*Cell*	821
*Antiviral Research*	8	18	2.6	950	*Journal of Medical Virology*	764
*Frontiers in Pharmacology*	8	11	4	132	*BMJ*	614
*International Journal of Infectious Diseases*	8	15	4	240	*Lancet Infectious Disease*	610
*Journal of Clinical Medicine*	8	18	4	367	*Antiviral Research*	607
*Journal of Medical Virology*	8	14	4	215	*Antimicrobial Agents and Chemotherapy*	596
*Open Forum Infectious Diseases*	8	10	4	131	*Lancet Respiratory Medicine*	535
*International Journal of Antimicrobial Agents*	7	9	3.5	1579	*Nature Communications*	534
*Science*	7	8	3.5	1311	*Cell Research*	521
*Viruses*	7	20	2.3	455	*Viruses*	485
*Clinical Microbiology and Infection*	6	9	3	293	*Proceedings of the National Academy of Sciences of the United States of America*	482
*EClinicalMedicine*	6	6	3	300	*Journal of Biological Chemistry*	473
*European Review for Medical and Pharmacological Sciences*	6	10	3	187	*Journal of Medicinal Chemistry*	458

**Table 3:** Top 20 most frequently globally cited documents.

Documents	DOI	Total Citations	TC per Year	Normalized TC
Holshue ML [27], 2020, *N Engl J Med*	10.1056/NEJMoa2001191	2997	1498.5	61.7045
Beigel JH [28], 2020, *N Engl J Med*	10.1056/NEJMoa2007764	2458	1229	50.6072
Wang Y [29], 2020, *Lancet*	10.1016/S0140-6736(20)31022-9	1575	787.5	32.4273
Grein J [30], 2020, *N Engl J Med*	10.1056/NEJMoa2007016	1420	710	29.236
Helms J [31], 2020, *Intensive Care Med*	10.1007/s00134-020-06062-x	1171	585.5	24.1094
Magro C [32], 2020, *Transl Res*	10.1016/j.trsl.2020.04.007	1025	512.5	21.1035
Geleris J [33], 2020, *N Engl J Med*	10.1056/NEJMoa2012410	918	459	18.9005
Wu C [34], 2020, *Acta Pharm Sin B*	10.1016/j.apsb.2020.02.008	905	452.5	18.6328
Sheahan TP [35], 2017, *Sci Transl Med*	10.1126/scitranslmed.aal3653	833	166.6	2.8314
Warren TK [36], 2016, *Nature*	10.1038/nature17180	742	123.67	3.5502
Agostini ML [37], 2018, *mBio*	10.1128/mBio.00221-18	736	184	4.1488
Mulangu S [38], 2019, *N Engl J Med*	10.1056/NEJMoa1910993	690	230	4.3671
Gao Y [39], 2020, *Sci*	10.1126/science.abb7498	592	296	12.1885
Pan H [40], 2021, *N Engl J Med*	10.1056/NEJMoa2023184	587	587	98.2945
Lescure FX [41], 2020, *Lancet Infect Dis*	10.1016/S1473-3099(20)30200-0	564	282	11.6121
Goldman JD [42], 2020, *N Engl J Med*	10.1056/NEJMoa2015301	545	272.5	11.2209
de Wit E [43], 2020, *Proc Natl Acad Sci USA*	10.1073/pnas.1922083117	485	242.5	9.9855
Wang F [44], 2020, *J Infect Dis*	10.1093/INFDIS/JIAA150	476	238	9.8002
del Valle DM [45], 2020, *Nat Med*	10.1038/s41591-020-1051-9	450	225.00	9.2649
Dashraath P [46], 2020, *Am J Obstet Gynecol*	10.1016/j.ajog.2020.03.021	448	224.00	9.2238

**Table 4:** Top 20 most frequently locally cited documents.

Documents	DOI	Year	Local Citations	Global Citations	LC/GC Ratio (%)
Berlin DA [47], 2020, *N Engl J Med*	10.1056/NEJMcp2009575	2020	940	398	236.18
Wang Y [29], 2020, *Lancet*	10.1016/S0140-6736(20)31022-9	2020	274	1575	17.40
Grein J [30], 2020, *N Engl J Med*	10.1056/NEJMoa2007016	2020	239	1420	16.83
Sheahan TP [35], 2017, *Sci Transl Med*	10.1126/scitranslmed.aal3653	2017	193	833	23.17
Warren TK [36], 2016, *Nature*	10.1038/nature17180	2016	178	742	23.99
Agostini ML [37], 2018, *mBio*	10.1128/mBio.00221-18	2018	160	736	21.74
Holshue ML [27], 2020, *N Engl J Med*	10.1056/NEJMoa2001191	2020	147	2997	4.90
Goldman JD [42], 2020, *N Engl J Med*	10.1056/NEJMoa2015301	2020	123	545	22.57
Mulangu S [38], 2019, *N ENGL J MED*	10.1056/NEJMoa1910993	2019	121	690	17.54
de Wit E [43], 2020, *Proc Natl Acad Sci USA*	10.1073/pnas.1922083117	2020	104	485	21.44
Spinner CD [48], 2020, *JAMA*	10.1001/jama.2020.16349	2020	94	409	22.98
Gao Y [39], 2020, *Sci*	10.1126/science.abb7498	2020	91	592	15.37
Yin W [49], 2020, *Sci*	10.1126/science.abc1560	2020	89	444	20.05
Geleris J [33], 2020, *N Engl J Med*	10.1056/NEJMoa2012410	2020	84	918	9.15
Wu C [34], 2020, *Acta Pharm Sin B*	10.1016/j.apsb.2020.02.008	2020	77	905	8.51
Tchesnokov EP [50], 2019, *Viruses*	10.3390/v11040326	2019	76	299	25.42
Brown AJ [51], 2019, *Antiviral Res*	10.1016/j.antiviral.2019.104541	2019	75	230	32.61
Pruijssers AJ [52], 2020, *Cell Rep*	10.1016/j.celrep.2020.107940	2020	70	162	43.21
Choy KT [53], 2020, *Antiviral Res*	10.1016/j.antiviral.2020.104786	2020	69	417	16.55
Williamson BN, 2020, *Nature*	10.1038/s41586-020-2423-5	2020	65	231	28.14

**Table 5:** Most cited countries with their total citations and average article citations.

Country	Total Citations	Average Article Citations
USA	26,602	39.82
China	7230	50.92
France	3047	50.78
India	2425	8.48
Italy	2103	14.91
United Kingdom	1383	26.6
Hong Kong	1278	75.18
Spain	1269	16.48
Canada	973	27.03
Switzerland	784	56
Germany	750	13.64
Egypt	575	19.17
Saudi Arabia	511	17.62
Singapore	472	67.43
Iran	373	6.11
Colombia	359	119.67
Korea	358	7.96
Pakistan	356	16.18
Australia	256	14.22
Denmark	233	14.56

**Table 6:** Most relevant affiliation and number of articles.

Affiliations	Articles
Icahn School of Medicine at Mount Sinai	103
Harvard Medical School	81
University of California	67
University of Michigan	53
University of Washington	49
All India Institute of Medical Sciences	47
Mayo Clinic	44
University of Milan	41
Shahid Beheshti University of Medical Sciences	38
Johns Hopkins University	35
Tehran University of Medical Sciences	35
Albert Einstein College of Medicine	34
Columbia University Irving Medical Center	33
The University of Hong Kong	33
Gilead Sciences	32
Massachusetts General Hospital	32
University of Utah	31
Columbia University	30
Wroclaw Medical University	30
Huazhong University of Science and Technology	28

## Data Availability

Raw and processed data are available upon request to the corresponding author.
